# Gdf11 regulates left‐right asymmetry development through TGF‐β signal

**DOI:** 10.1111/cpr.13765

**Published:** 2024-10-15

**Authors:** Wantao Yao, Zhaohui Wei, Xinning Tian, Jin Tan, Jingwen Liu

**Affiliations:** ^1^ School of Basic Medicine Anhui Medical University Hefei China; ^2^ College of Animal Science and Technology Nanjing Agricultural University Nanjing China

## Abstract

During the embryonic developmental stage in vertebrates, internal organs are arranged along the left–right axis. Disruptions in this process can result in congenital diseases or laterality disorders. The molecular mechanisms of left–right asymmetry in vertebrate development remain largely unclear. Due to its straightforward structure, zebrafish has become a favoured model for studying early laterality events. Here, we demonstrate that growth and development factor 11 (Gdf11) is essential for left–right development via TGF‐β signalling. Morphological analysis showed that *gdf11* morphants and mutants displayed clear heart and liver laterality disorders in a Nodal signal‐dependent manner. Additionally, we found that Kupffer's vesicle formation and ciliogenesis were impaired following *gdf11* deletion. We also observed that Gdf11 may form a heterodimer with Spaw, which promotes Smad2/3 phosphorylation and activates TGF‐β signalling. Subsequently, Gdf11 promotes left–right laterality by stimulating Foxj1a and its target gene expression. In summary, we reveal a critical role of Gdf11 in left–right patterning, providing fundamental insights into the developmental process of left–right asymmetry.

## Graphical abstract

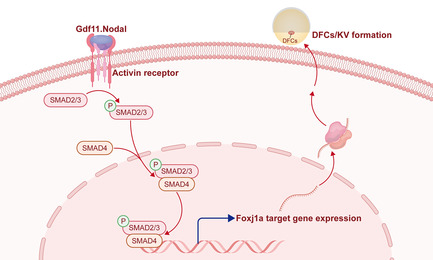

## INTRODUCTION

In vertebrates, visceral organs are asymmetrically positioned along the left–right axis. Disruptions in the development of laterality result in misplaced internal organs and abnormal morphogenesis, such as *situs inversus totalis* or heterotaxy.^1^ The bilateral symmetry is disrupted by the left–right organizer (LRO) during early embryonic stages.^2^ Within the LRO, motile cilia rotate counter‐clockwise, generating a “Nodal flow.”^3^ This flow creates a gradient of a putative morphogen, establishing differences between the left and right lateral plate mesoderm. The LRO is referred to as Kupffer's vesicle in zebrafish and as the node in mammals.^4^ Kupffer's vesicle is a transient organ at the end of the notochord, originating from a group of cells known as “dorsal forerunner cells (DFCs).” DFCs proliferate during the epiboly stage and differentiate into ciliated epithelial KV cells from the bud stage.^5^ Several signalling pathways, including Wnt/β‐Catenin, FGF, TGF‐β, and chemokine signals, have been implicated in this process.^5^, ^6^, ^7^, ^8^

Gdf11 is a member of the transforming growth factor‐beta (TGF‐β) ligand family.^9^ It has been widely studied for its roles in anti‐aging, aplastic anaemia, dental diseases, kidney development/fibrosis, and hypertrophic cardiomyopathy.^10^ In zebrafish, knocking down *gdf11* affects liver development through histone deacetylase 3.^11^, ^12^ Knocking down *gdf11* with MO led to a clear caudal shift of *hoxc10a* expression and pelvic fin displacement.^13^ Additionally, *gdf11* mutants exhibit obvious craniofacial and body axis developmental defects.^14^ Recently, *gdf11* expression has been detected in Kupffer's vesicle.^15^ However, the impact of *gdf11* on left–right asymmetry development remains unclear.

This study provides clear evidence that Gdf11 is essential for L‐R asymmetry patterning. *gdf11* ^−/−^ mutants displayed significant left–right asymmetry development defects and reduced DFC proliferation. Ciliogenesis in KV was also impaired following *gdf11* deletion. *southpaw* is the homologous gene of Nodal in zebrafish and is necessary for visceral organ left–right asymmetry development.^16^ Subsequent biochemical and functional experiments suggested that Gdf11 may form a heterodimer with Spaw, which activates TGF‐β signalling and the expression of cilia‐related genes, including *foxj1a* and its target genes. We further used a small molecule activator of TGF‐β signal to rescue DFC proliferation and cilia formation upon *gdf11* deletion. In summary, we identified a novel role of *gdf11* in laterality development and revealed the molecular mechanism by which *gdf11* influences left–right asymmetry development. Furthermore, our research offers a theoretical basis for genetic counselling and molecular diagnosis of LR asymmetry‐related diseases.

## 1 MATERIALS AND METHODS

### 1.1 Zebrafish strains

The zebrafish line used in this study is the AB strain. Embryos were maintained in Holtfreter's solution at 28.5°C and staged based on their morphology. Homozygous *gdf11* ^−/−^ mutants were identified through genotyping. The primer used for *gdf11* ^−/−^ mutant identification are Forward primer: 5′‐CTGAACCTCTGGTGCAGGTG‐3′; Reverse primer: 5′‐CAGTCCCTCCTCTCCAGGC‐3′. *Tg(sox17)* transgenic embryos were used to indicate DFC and KV cells during development.

### 1.2 Whole‐mount in situ hybridization

Whole‐mount in situ hybridization was performed using the NBT‐BCIP substrate following standard procedures.^5^ Specific primers used to amplify *gdf11*, *cmlc2*, *hhex*, *spaw*, *pitx2*, *ntl*, *lefty1*, *foxj1a*, *lrdr1*, *spag6*, and *efhc1* are listed in Table S1.

### 1.3 Morpholinos and microinjection

*gdf11* and control MOs were synthesized by Gene Tools (Philomath, USA) and diluted in nuclease‐free water to a concentration of 1 mM. The *gdf11* antisense MO sequence was 5′‐ATACCTTTTCATGTTGTTAAATATC‐3′,^12^ and the 5 bp mismatch control MO sequence was 5′‐ATAGCTTTTGATCTTCTTAAAAATC‐3′. The zebrafish *gdf11* gene was knocked down using *gdf11* MO (4 ng/embryo).

### 1.4 Alcian Blue staining

Embryos at 120 hpf were fixed with 4% paraformaldehyde overnight. After that, embryos were washed in distilled water for 8 h. The embryos were then stained with Alcian Blue staining buffer (0.015% Alcian Blue, 80% ethanol, and 20% acetic acid) for 12 h at room temperature and then de‐stained with graded ethanol. Next, the embryos were digested with 0.5% trypsin in supersaturated borax at room temperature for 5 h. The embryos were then transferred to 1% KOH and dehydrated with a graded series of glycerol.

### 1.5 Cell lines and transfection

HEK 293T cells (American Tissue Culture Collection, ATCC, USA) were cultured in DMEM medium supplemented with 10% FBS (HyClone, Thermo Fisher Scientific) and 1% Penicillin/Streptomycin (Gibco) in a 37°C humidified incubator with 5% CO_2_. Transfections were performed using Lipofectamine 3000 (L3000015, Invitrogen) according to the manufacturer's instructions.

### 1.6 Immunostaining and confocal microscope

Embryos were fixed in 4% paraformaldehyde overnight, rinsed with PBST three times for 10 min each, and blocked at room temperature for 1 h in 10% heat‐inactivated goat serum. They were then stained with primary antibodies overnight at 4°C: anti‐acetylated‐Tubulin (1:400; T6793, Sigma), anti‐α‐pH3 (1:200, ab80612, Abcam), anti‐α‐PKC (1:200, sc‐216, Santa Cruz Biotechnology), and anti‐GFP (1:1000; A‐11122, Invitrogen). Samples were washed with PBST and incubated with secondary antibodies, including DyLight 488‐conjugated goat anti‐rabbit IgG (1:200; 711‐545‐152, Jackson), DyLight 594‐conjugated goat anti‐mouse IgG (1:200; 715‐585‐150, Jackson), DyLight 488‐conjugated AffiniPure goat anti‐mouse IgG (1:200; 715‐545‐150, Jackson), and DyLight 594‐conjugated AffiniPure goat anti‐rabbit IgG (1:200; 711‐585‐152, Jackson) for 2 h at room temperature. DAPI (1:10,000, Sigma) was used for nuclear staining.

### 1.7 TUNEL staining

Zebrafish embryos were fixed and stained using a TUNEL staining kit (12156792910, Roche) according to the manufacturer's instructions.

### 1.8 Pharmacological treatment

To inhibit TGF‐β activity, embryos were treated with 0.5 μM SB431542 (HY‐10431, MedChemExpress) from the shield stage to the 75% epiboly or 10 ss. For activating TGF‐β activity, mutant embryos were treated with 0.2 μM SRI‐011381 (HY‐100347, MedChemExpress) from the shield stage to the 75% epiboly or 10 ss.

### 1.9 Immunoblotting and immunoprecipitation assays

For immunoblotting, we used the following affinity‐purified antibodies: Anti‐Flag (1:5000; F2555, Sigma), anti‐Myc (1:3000; M047‐3, MBL), anti‐Gdf11 (1:300, sc‐81952, Santa Cruz), anti‐Foxj1 (1:200; ab220028, Abcam), anti‐p‐ALK5 (1:1000, Affinity, Cat. No. AF‐3489), anti‐p‐Smad2 (1:1000, Santa Cruz, sc‐101801), anti‐Smad2 (1:1000, Santa Cruz, sc‐393312), anti‐Smad4 (1:1000, Invitrogen, PA5‐34806), and anti‐β‐Tubulin (1:5000, CW0098M, CWBIO).

For co‐immunoprecipitation assays, embryos or HEK293T cells were harvested and lysed with RIPA buffer (R0010, Solarbio) containing a protease inhibitor mixture. Lysates were incubated with anti‐Flag‐agarose beads (A2220, Sigma) at 4°C overnight. Beads were washed four times with RIPA buffer, and the supernatants were separated by SDS‐PAGE and visualized by western blotting.

### 1.10 Quantitative real‐time PCR (qRT‐PCR)

Total RNA was isolated from 50 WT or mutant embryos using the Total RNA Extraction Kit (R1200, Solarbio). Three micrograms of total RNA was transcribed into cDNA using the miScript II RT‐Kit (218161, Qiagen) for mRNA detection. qRT‐PCR analysis was performed using the TaqMan One Step RT‐qPCR Kit (T2210, Solarbio). Gene expression changes were quantified using the delta–delta‐Ct method. Primers used are listed in Table S2.

### 1.11 Statistical analysis

The KV lumen size and cilia number and length were measured using ImageJ software. All results were expressed as the mean ± SD. Differences were analysed using the unpaired two‐tailed Student's *t*‐test. Results were considered statistically significant at *p* < 0.05, with “*” indicating *p* < 0.05, “**” indicating *p* < 0.01, and “***” indicating *p* < 0.001, “****” indicating *p* < 0.0001. Data were analysed using GraphPad Prism 7 software.

## 2 RESULTS

### 2.1 Gdf11 has evident expression in DFCs and Kupffer's vesicle

**FIGURE 1:**
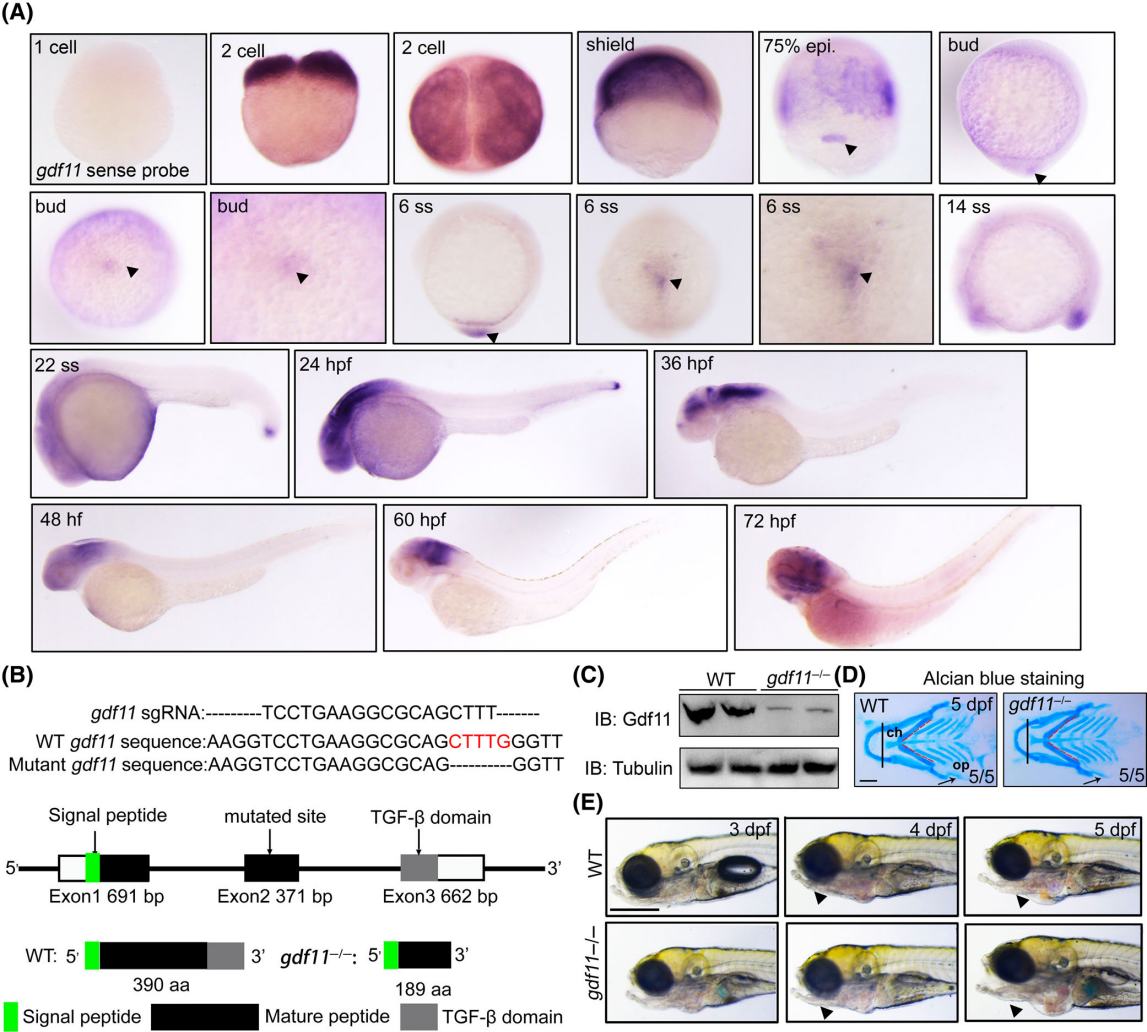
*gdf11* expression pattern and mutant construction. (A) Whole‐mount in situ hybridization with *gdf11* from the 2‐cell stage to 72 hpf. *gdf11* sense probe was utilized as negative control at 1 cell stage. (B) Schematic picture showing the wild type and mutated site of *gdf11* ^−/−^ mutants. (C) Western blots of lysates from WT and *gdf11* ^−/−^ embryos using antibodies against Gdf11 with β‐tubulin as a loading control. (D) Alcian blue staining in both WT and *gdf11* mutants. *gdf11* mutants exhibit defects in the alignment of upper and jaw elements, the angle of ch. articulation, and the morphology of the op. The defects of the alignment of upper/jaw elements and the morphology of the op in *gdf11* mutant were marked with straight lines and arrows in the picture. op，opercle; ch，ceratohyal. Scale bar, 50 μm. (E) Live images of WT and *gdf11* ^−/−^ mutants from 3 to 5 dpf. Black triangle indicates craniofacial cartilage. Scale bar, 100 μm.

Previous research has indicated that *gdf11* expression is activated in Kupffer's vesicle and at the tail tip at the 10‐somite stage (ss).^15^ To further clarify the specific expression patterns of *gdf11*, we conducted whole mount in situ hybridization (WISH) at early embryonic stages in zebrafish. As shown in Figure 1A, *gdf11* expression was detected at the 2‐cell stage, indicating it is a maternally expressed gene. From the epiboly stage, *gdf11* was activated in the DFCs and developing Kupffer's vesicle (Figure 1A). Based on these observations, we hypothesized that *gdf11* might play an essential role in Kupffer's vesicle formation and left–right patterning.

Considering the expression pattern of *gdf11*, we decided to test its effect on left–right patterning. Gdf11 consists of three domains: the signal peptide, the mature proprotein, and the TGF‐β domain.^17^ Using CRISPR/Cas9 technology, we designed a sgRNA targeting exon 2 of *gdf11*, resulting in a 5 bp deletion mutant that produced a premature stop codon (Figure 1B). Western blot analysis with a Gdf11 antibody showed a significant decrease of Gdf11 protein levels in *gdf11* mutants, and the weak bands in mutant lysates probably were due to the protein residues caused by multiple transcripts or alternative splicing of *gdf11* (Figure 1C). Consistent with previous findings.^14^ the *gdf11* mutants we created exhibited noticeable defects in craniofacial cartilage formation, including alignment of upper and jaw elements, the angle of ch. articulation, and the morphology of the op. (Figures 1D,E and S1). In summary, we identified that *gdf11* is clearly expressed in DFCs/Kupffer's vesicle at early stages and we successfully generated *gdf11* homozygous mutants.

### 2.2 Ablation or knockdown of *gdf11* compromises L‐R laterality

**FIGURE 2:**
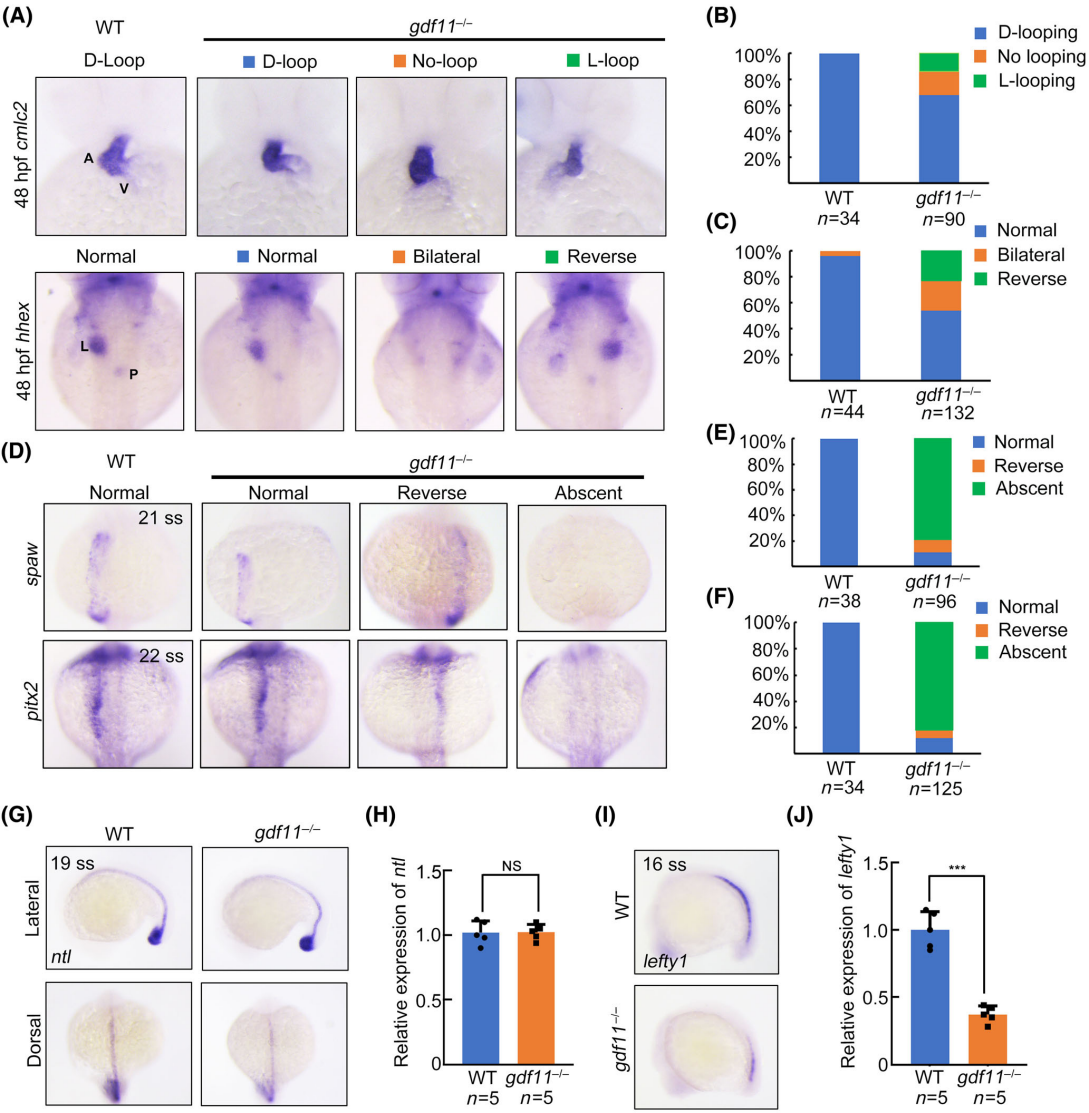
*gdf11* is essential for left–right patterning. (A) Cardiac looping and liver/pancreas positions analysed at 48 hpf. Heart looping visualized by in situ hybridization with *cmlc2* probe at 48 hpf (top panel). A, atrium; V, ventricle. Liver/pancreas positions visualized by in situ hybridization with *hhex* probe at 48 hpf (bottom panel). L, liver; P, pancreas. (B) Graph showing the percentage of embryos with normal D‐looping (dark blue), no looping (yellow), or L‐looping (green). (C) Graph showing the percentage of embryos with normal liver/pancreas position (dark blue), bilateral liver/pancreas position (yellow), or reverse liver/pancreas position (green). (D) *spaw* (upper panel) and *pitx2* (lower panel) expression in WT and *gdf11* ^−/−^ mutants at 21 or 22 ss. (E, F) Percentage of *spaw* (E) and *pitx2* (F) expression in WT and *gdf11* ^−/−^ mutants. Normal (dark blue), reverse (yellow), or absent (green). (G, H) Graphs depicting the expression of midline *ntl* at 19 ss (G) and the percentage of *ntl* expression in WT and *gdf11* ^−/−^ mutants (H). (I, J) Graphs depicting the expression of *lft1* at 16 ss (I) and the percentage of *lft1* expression in WT and *gdf11* ^−/−^ mutants (J).

The earliest morphological sign of left–right asymmetry is the leftward jogging of the atrioventricular canal, followed by the dextral looping of the heart tube^18^ To investigate the left–right patterning in *gdf11* mutants, we conducted WISH experiments using a cardiac myosin light chain 2 (*cmlc2*) probe at 30 h post‐fertilization (hpf) and 48 hpf. In wild type (WT, *gdf11* ^+/+^) embryos, normal jogging and looping to the right were observed, whereas approximately 35% of *gdf11* mutants exhibited defects in jogging and looping (Figures 2A,B and S1). We next analysed the expression of *hhex* in WT and *gdf11* ^−/−^ embryos to check the positioning of liver and pancreas. The positions of these organs were also disrupted in *gdf11* ^−/−^ mutants (Figure 2A,C). Additionally, in situ hybridization against *cmlc2* and *hhex* was performed on embryos injected with control or validated *gdf11* MO.^12^ We found knockdown of *gdf11* also resulted in left–right asymmetry developmental defects (Figure S2).

Left–right asymmetry is regulated by conserved Nodal signal in vertebrates, with Nodal and Lefty (TGF‐β‐related proteins) being asymmetrically expressed and playing critical roles in L‐R asymmetry patterning. *pitx2c* is the target gene of *southpaw* during left–right asymmetry signal transduction and instructs visceral organ LR asymmetry development.^4^ Therefore, we performed in situ hybridization with *spaw* and *pitx2c* probes in both WT and *gdf11* mutants at somite stage.^3^ WT embryos showed normal *spaw* and *pitx2c* expression in the left LPM, whereas a significant portion of *gdf11* mutants had absent *spaw* and *pitx2c* expression with few embryos had the expressions in the right LPM (Figure 2D–F). The intact dorsal midline acts as a barrier preventing the diffusion of asymmetric morphogens between the L‐R sides.^2^ Considering this, we examined *no tail* (*ntl*) expression and performed qRT‐PCR, finding that the midline barrier was intact in both WT and *gdf11* mutants (Figure 2G,H). However, the expression of the molecular barrier marker *lefty1* was reduced in the mutants (Figure 2I,J).^19^ Based on these observations, we concluded that *gdf11* is necessary for left–right patterning in a Nodal signal‐dependent manner.

### 2.3 Absence of *gdf11* attenuates Kupffer's vesicle lumen size and cilia formation

**FIGURE 3:**
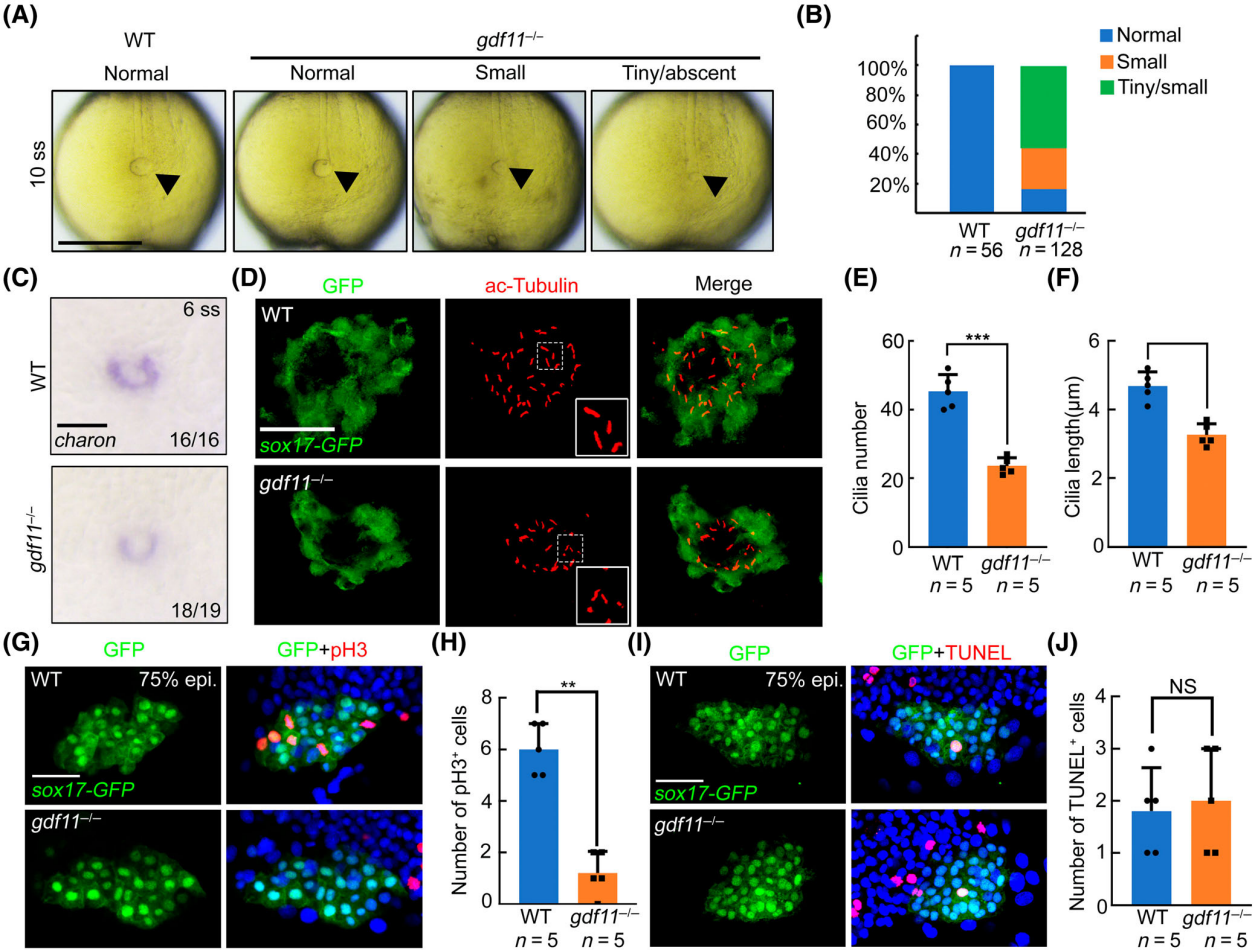
*gdf11* is required for DFC proliferation and KV cilia formation. (A) Live image of Kupffer's vesicle at the 10‐somite stage in both WT and *gdf11* ^−/−^ mutants. Scale bar, 200 μm. (B) Percentage of KV size in WT and *gdf1* ^−/−^ mutants. (C) in situ hybridizations of *charon* were used to assess Kupffer's vesicle lumen size at 6 ss. Scale bar, 100 μm. (D) Visualization of KV cilia at the 10‐somite stage using an anti‐acetylated tubulin antibody in control or *gdf11* ^−/−^ embryos of the *Tg(sox17‐GFP)* line, showing both KV cells (green) and cilia (red). Scale bar, 50 μm. (E,F) Number (E) and length (F) of KV cilia in WT and mutant embryos. Graph indicates the mean of cilia number and length, error bars represent the standard deviation. Statistical significance between WT and *gdf11* ^−/−^ mutants was determined using a two‐tailed unpaired *t*‐test. (G, H) Confocal images of 75% epiboly zebrafish embryos with pH3 staining in a *Tg(sox17‐GFP)* background (G). Quantification of pH3 positive cells (H). Error bars represent the standard deviation. Statistical significance was determined using a two‐tailed unpaired *t*‐test. Scale bar, 50 μm. (I, J) Confocal images of 75% epiboly zebrafish embryos with TUNEL staining in a *Tg(sox17‐GFP)* background (I). Quantification of TUNEL positive cells (J). Error bars represent the standard deviation. Statistical significance was determined using a two‐tailed unpaired *t*‐test. Scale bar, 50 μm.

Left–right asymmetry patterning in zebrafish is established by Kupffer's vesicle, which is analogous to the mammalian node.^20^ To investigate the effect of *gdf11* deletion on Kupffer's vesicle, we first examined the lumen size of Kupffer's vesicle in WT and *gdf11* mutant embryos at the 10‐somite stage (ss). Many *gdf11* mutants showed a smaller or reduced KV lumen size compared to WT embryos (Figures 3A,B and S3A). We also assessed KV lumen size through WISH against *charon*, confirming that the KV lumen size reduction in *gdf11* ^−/−^ mutants (Figures 3C and S3B). We then tracked DFCs and KV morphogenesis in WT and *gdf11* ^−/−^ in a *Tg(sox17‐GFP)* background. The DFC morphology remained unchanged at 75% epiboly and bud stages in mutants, indicating that the induction and clustering of DFCs are unaffected by *gdf11* deletion. However, KV lumen size was significantly reduced in *gdf11* ^−/−^ at 6 ss compared to control embryos (Figure S3C). Cell polarity, examined using the α‐PKC antibody, was also unchanged in both WT and *gdf11* ^−/−^ mutants after *gdf11* deletion (Figure S3D).

Motile cilia in KV rotate counter‐clockwise, activating *spaw* and *pitx2c* expressions in the left LPM and initiating left–right asymmetric development.^2^ We assessed KV cilia by probing acetylated tubulin in the *Tg(sox17‐GFP)* background at 10 ss. In comparison to control embryos, the number and length of cilia were significantly reduced in *gdf11* ^−/−^ mutants (Figure 3D–F).

Observations of KV lumen size and cell morphology suggest that the number of KV cells is reduced in *gdf11* mutants. To explore the effect of *gdf11* on cell number, we performed pH3 staining in both WT and *gdf11* ^−/−^ embryos at 75% epiboly, as vigorous proliferation occurs in DFCs during epiboly stages.^5^ We found a significant decrease of DFC proliferation upon *gdf11* deletion (Figure 3G,H). Additionally, cell death was assessed through TUNEL staining at 75% epiboly, revealing no significant change in cell death between WT and *gdf11* mutants (Figure 3I,J). These analyses provide strong evidence that *gdf11* is crucial for DFC proliferation and KV ciliogenesis in zebrafish.

### 2.4 Gdf11 controls dfc proliferation and cilia formation through TGF‐β signalling

Previous studies have shown that Gdf1 forms a heterodimer with Nodal, which activates Nodal signal and endoderm differentiation, with Tbx6 upstream of Gdf1 regulating LR patterning.^21^, ^22^ Based on this, we hypothesized that Gdf11 might also form a heterodimer with Spaw and activate TGF‐β signal.

**FIGURE 4:**
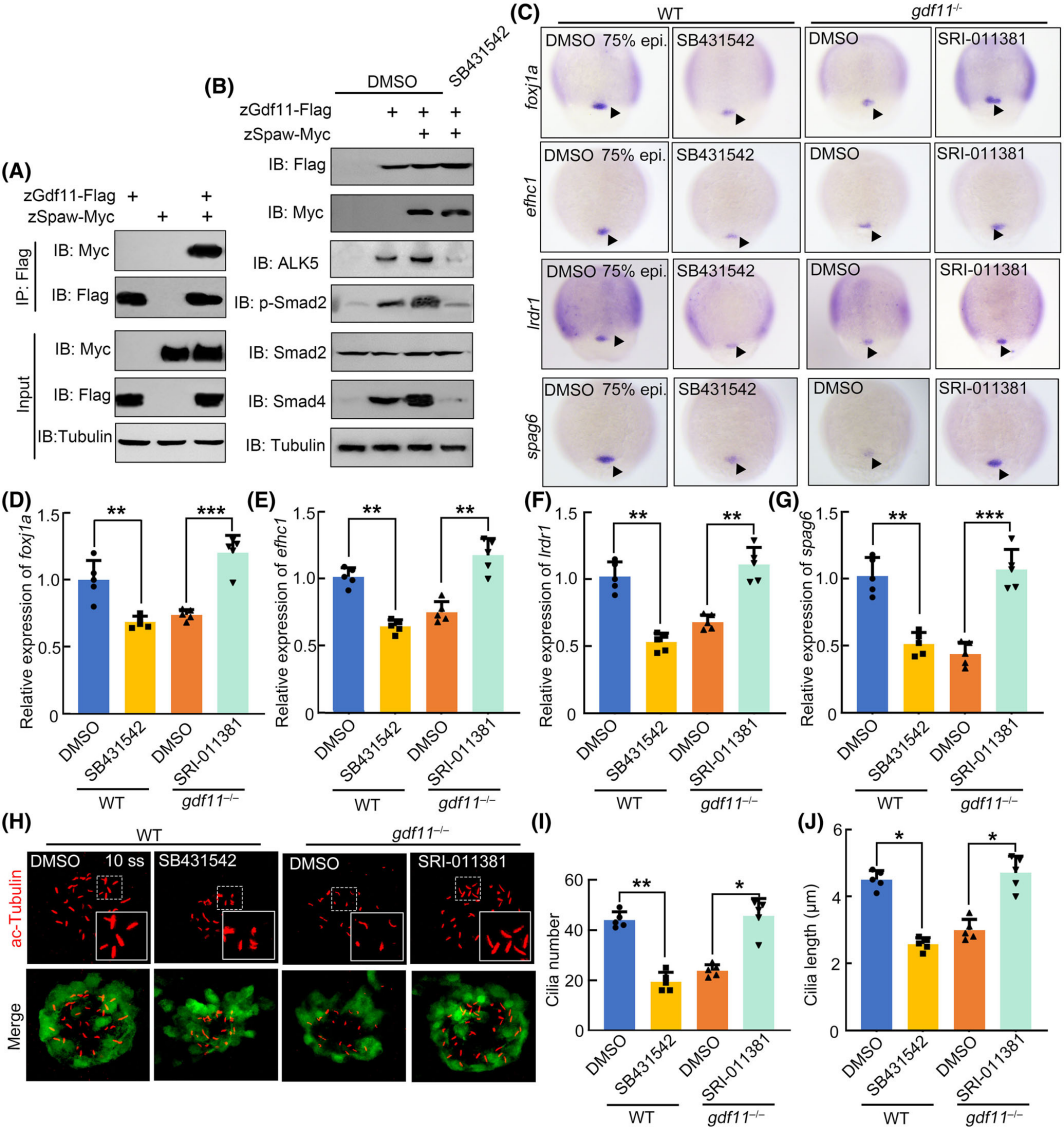
Gdf11 forms heterodimer with Spaw and regulates DFC specification through TGF‐β signal. (A) Immunoprecipitation between zGdf11‐Flag and zSpaw‐Myc in HEK 293T cells. (B) Western blot results of overexpressed zGdf11‐Flag and zSpaw‐Myc in HEK 293T cells with or without SB431542 treatment. (C) *foxj1a* and its target gene expression using WISH in WT or *gdf11* mutants with or without treatment. (D–G) Expression levels of *foxj1a* and its target genes. Error bars represent the standard deviation. Statistical significance was determined using a two‐tailed unpaired *t*‐test. (H) Visualization of KV cilia at the 10‐somite stage using an anti‐acetylated tubulin antibody in both WT or *gdf11* ^−/−^ mutants with or without SB431542/SRI‐011381 treatment. Scale bar, 50 μm. (I, J) Number (I) and length (J) of KV cilia in WT and mutant embryos with or without SB431542/SRI‐011381 treatment. Graph indicates the mean of cilia number and length, error bars represent the standard deviation.

To test this hypothesis, we conducted immunoprecipitation between zGdf11‐Flag and zSpaw‐Myc in HEK 293T cells. The results showed that Gdf11 has interaction with Spaw (Figure 4A). To further confirm the interaction, we performed native‐PAGE in HEK 293T cells transfected with zGdf11‐Flag and zSpaw‐Myc. The protein size observed in native‐PAGE was significantly larger than its size in SDS‐PAGE (Figure S4A). GDF11 binds to activin receptor I (ActRI) and activin receptor II (ActRII), subsequently activating downstream pathways with kinase functions. GDF11 regulates gene expression in various tissues by activating SMAD and non‐SMAD pathways.^23^, ^24^, ^25^ Next, we investigated whether the dimer activates TGF‐β signal. As shown in Figure 4B, the levels of ALK5, p‐Smad2, and Smad4 increased obviously when Gdf11 was overexpressed. These protein levels were further elevated when Gdf11 and Spaw were co‐expressed. SB431542, a well‐known small molecule inhibitor of TGF‐β, binds specifically to activin receptor‐like kinase receptors (including ALK5, ALK4, and ALK7) and inhibits Smad2/3 activation, thereby blocking TGF‐β signal transduction.^26^ SB431542 treatment eliminated the increases in ALK5, p‐Smad2, and Smad4 induced by the overexpression of Gdf11 and Spaw (Figure 4B). Foxj1a is the master regulator of ciliogenesis in KV.^27^, ^28^ We observed that Foxj1 protein level increased with Gdf11 overexpression, and this increase was even higher when Gdf11 and Spaw were co‐overexpressed. However, SB431542 treatment negated this increase (Figure S4B). Considering the defective cilia formation and the activation of TGF‐β signal by the Gdf11.Spaw dimer, we treated WT and mutant embryos with SB431542 or SRI‐011381 (a commonly used TGF‐β activator) from the shield stage to 75% epiboly.^5^ Afterward, these embryos were collected for WISH against *foxj1a* and its target genes (*efhc1*, *lrdr1*, and *spag6*).^27^ WT embryos treated with SB431542 exhibited phenotypes similar to *gdf11* mutants, showing reduced *foxj1a* and target gene expressions. We also observed that mutant embryos treated with SRI‐011381 showed significant recovery of the expression levels of *foxj1a* and its target genes (Figure 4C). Further validation using qRT‐PCR confirmed the relative expression of *foxj1a* and its target genes in WT or *gdf11* mutants treated with the inhibitor or agonist (Figure 4D–G). Additionally, we examined the number and length of cilia in KV after treatment with SB431542 or SRI‐011381. As shown in Figure 4H–J, SB431542 treatment in WT embryos led to a decrease in cilia number and length, resembling the *gdf11* mutants. Conversely, SRI‐011381 treatment restored cilia morphology in the mutants. These findings suggest that Gdf11 promotes L‐R patterning by activating TGF‐β signalling and downstream *foxj1a* expression.

## 3 DISCUSSION AND CONCLUSION

**FIGURE 5:**
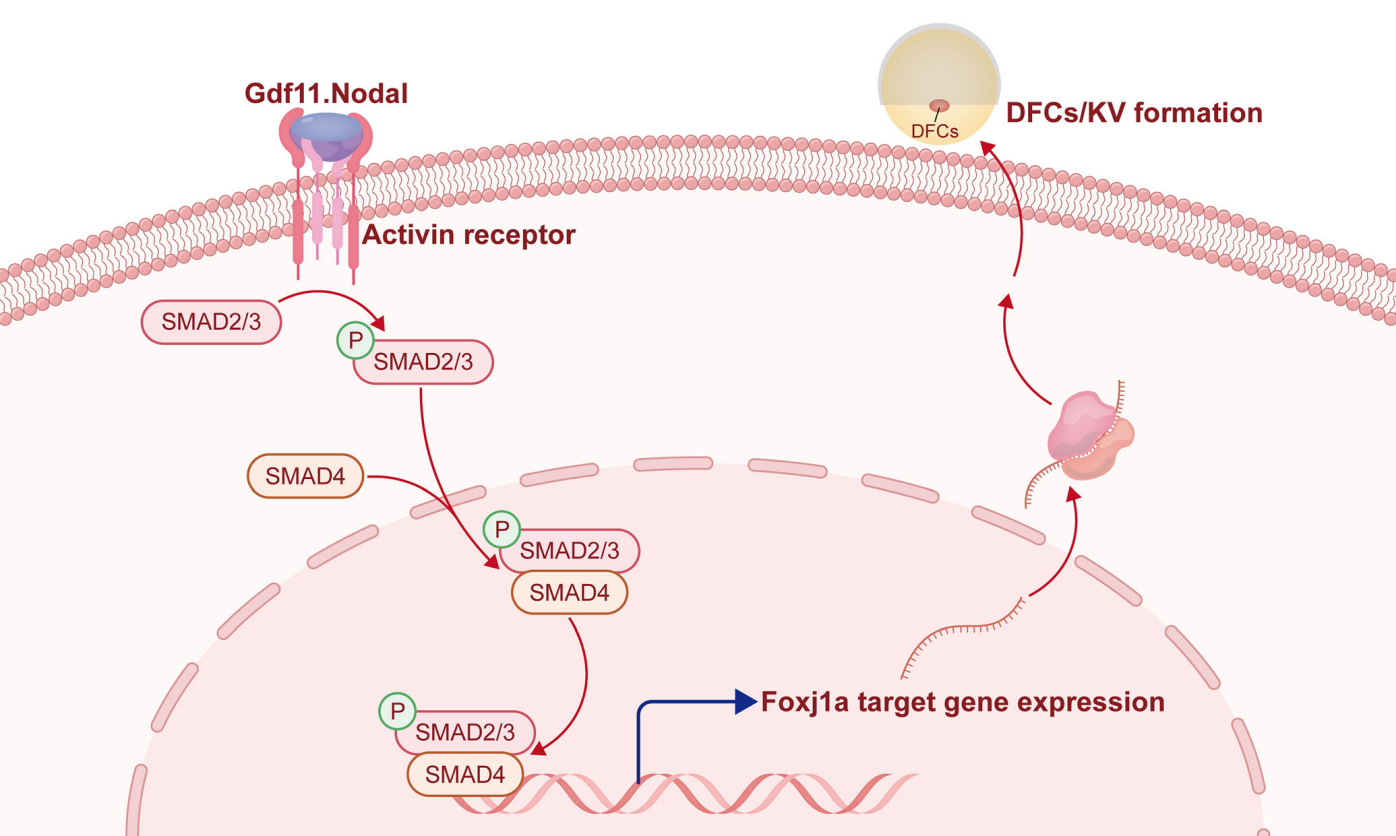
Mechanism diagram of Gdf11 regulating left–right asymmetry development. Gdf11 forms a heterodimer with Spaw, activating the activin receptor and phosphorylating Smad2/3. This process DFC proliferation, subsequently activates the expression of *foxj1a* and its target genes, promoting cilia formation. The diagram was drawn using Adobe Illustrator 2020 software.

Our study demonstrated that *gdf11* regulates left–right asymmetry development through TGF‐β signal. The DFC proliferation and ciliogenesis are both reduced upon *gdf11* deletion. Gdf11 is essential for the expression of *foxj1a* and its target genes, which are crucial for the asymmetry development and formation of DFCs/KV (Figure 5). Additionally, treatment with a TGF‐β signal activator rescued DFC proliferation and KV cilia formation after *gdf11* deletion.

In vertebrates, Gdf11 is expressed in various tissues, including the liver, kidneys, heart, pancreas, intestines, skeletal muscles, and nervous system.^9^ During the early embryonic stage in mice, Gdf11 is primarily expressed in the primitive streak and tail bud regions.^29^ Previous studies and our results also confirmed the expression of *gdf11* in KV.^15^ Due to the lethality of homozygous *Gdf11* ^−/−^ mutants in mice, understanding the role of Gdf11 in left–right asymmetry patterning development in mammal is challenging.^29^ However, by taking advantage of the application of CRISPR / Cas9 technology in zebrafish, we successfully constructed zebrafish *gdf11* ^−/−^ mutants and discovered that Gdf11 promotes LR patterning by activating Nodal signal.

Recently, *Gdf1* was identified as a Nodal co‐ligand necessary for the long‐range action of Nodal, and *Gdf1* was also found to be a downstream target of Tbx6.^22^, ^30^ The Gdf1 transgene partially rescues laterality defects in zebrafish.^30^ In most Gdf1^−/−^ or Nodal^−/−^ embryos, the expression of left LPM‐specific genes (including *lefty1*, *nodal*, and *Pitx2*) was absent, suggesting that Gdf1 and Nodal act upstream of these genes.^30^ In our study, the expression of *spaw*, *pitx2*, and *lefty1* was also absent, indicating that Gdf11 acts upstream of these genes to activate their expression. Based on these observations, we hypothesized that Gdf11 may also form a heterodimer with Spaw to regulate left–right asymmetry. Co‐expression of Gdf11 and Spaw also increases the phosphorylation of Smad2/3, Smad4, and Foxj1 protein levels. As a member of the GDF family, we found that *Gdf11* may also act as a co‐ligand of Spaw in zebrafish.

## AUTHOR CONTRIBUTIONS

J.W.L. supervised and designed the project. W.T.Y. and Z.H.W. were responsible for drafting the manuscript. X.N.T. and J.T. conducted data analysis. W.T.Y. and Z.H.W. performed the experiments. All authors actively contributed to the article and approved the submitted version.

## FUNDING INFORMATION

This work was supported by the National Natural Science Foundation of China (32300681), Anhui Province Scientific Research Plan Project (2023AH050657), and Scientific research start‐up funds of Anhui Medical University (0101045201).

## CONFLICT OF INTEREST STATEMENT

The authors declare no conflict of interest.

## Supporting information


Figure S1.

Table S1.

Table S2.

## Data Availability

The data that support the findings of this study are available from the corresponding author upon reasonable request.
