# Supplementary material for: Gdf11 regulates left‐right asymmetry development through TGF‐β signal
Source: Cell Prolif. 2024 Oct 15;58(3):e13765. doi: 10.1111/cpr.13765 (PMC11882743; doi:10.1111/cpr.13765)

**Supporting Figures**


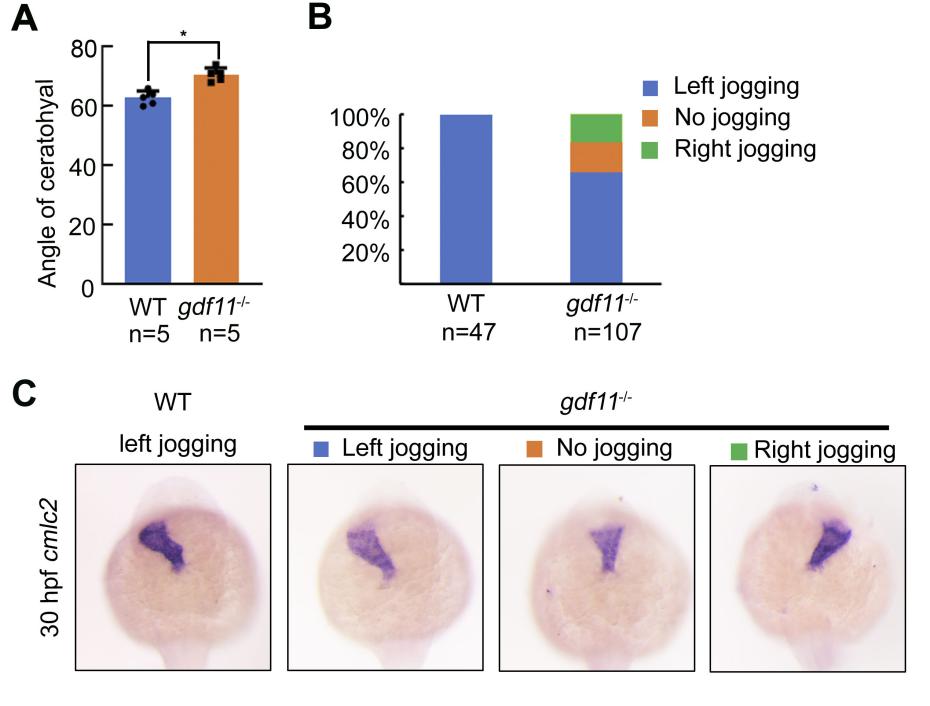


**Figure S1 *gdf11* effects cartilage formation and heart jogging in zebrafish.** (A) Graph showing the angle of ch. articulation in both WT and *gdf11*^-/-^ embryos. (B) Graph showing the percentage of embryos with normal left jogging (dark blue), no jogging (yellow), or right jogging (green). (C) Heart jogging was examined using WISH with the *cmlc2* probe in both WT and mutant embryos at 30 hpf.

**
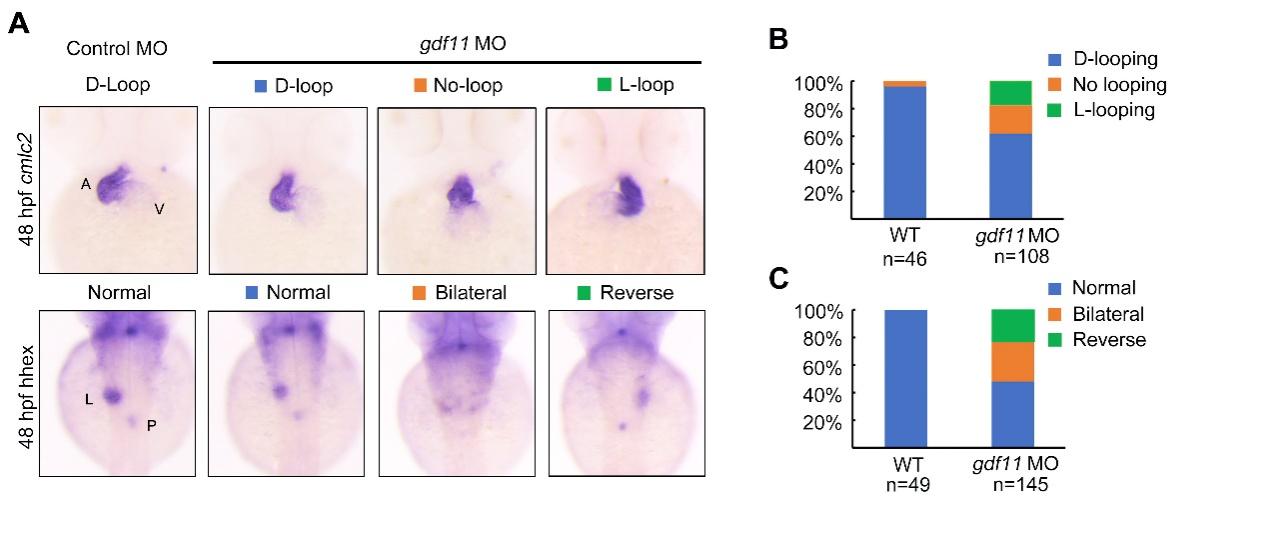
**

**Figure S2 *gdf11* knockdown leads to laterality defects in zebrafish embryos.** (A-C) Representative images showing heart asymmetry labeled by *cmlc2* and liver position labeled by *hhex* at 48 hpf in embryos injected with control MO or *gdf11* MO (A), with quantification (B and C).


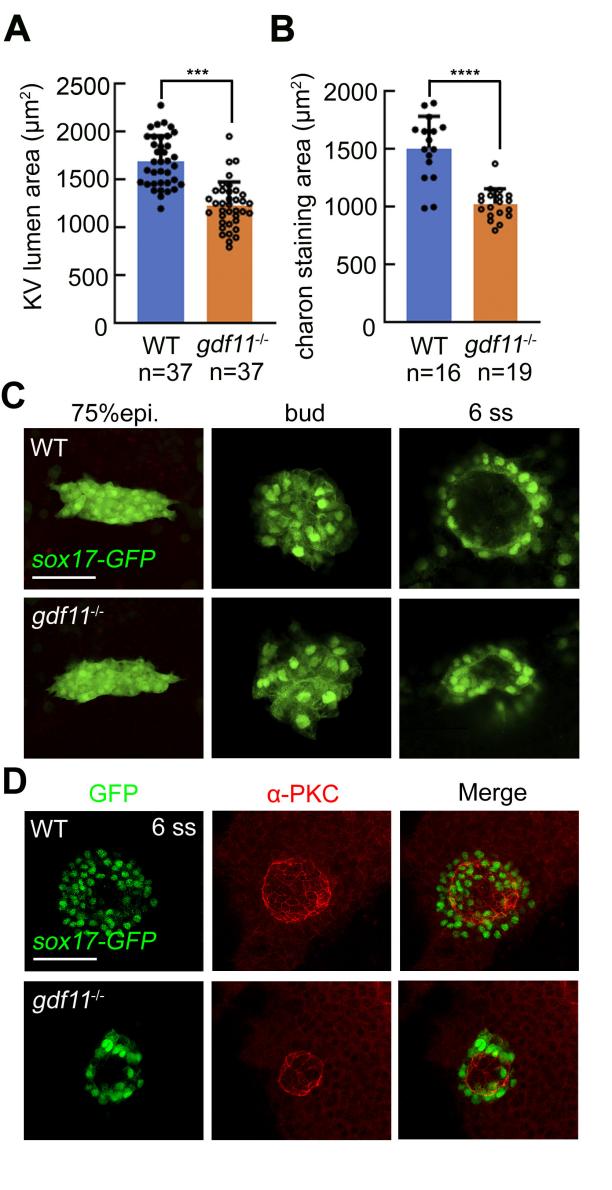


**Figure S3 *gdf11* is necessary for KV modelling.** (A) Statistical analysis of KV lumen size in WT and *gdf11*-/- embryos at 10 ss. (B) Statistical analysis of *charon* positive lumen area in WT and *gdf11*^-/-^ embryos at 10 ss. (C) Live images of DFC morphogenesis in WT and *gdf11*^-/-^ mutants in a *Tg(sox17-GFP)* background. Scale bar, 100 µm. (D) Immunofluorescence staining against GFP (green) and α-PKC (red) antibodies in WT and *gdf11* ^-/-^ mutant embryos at 6 ss. Scale bar, 100 µm.


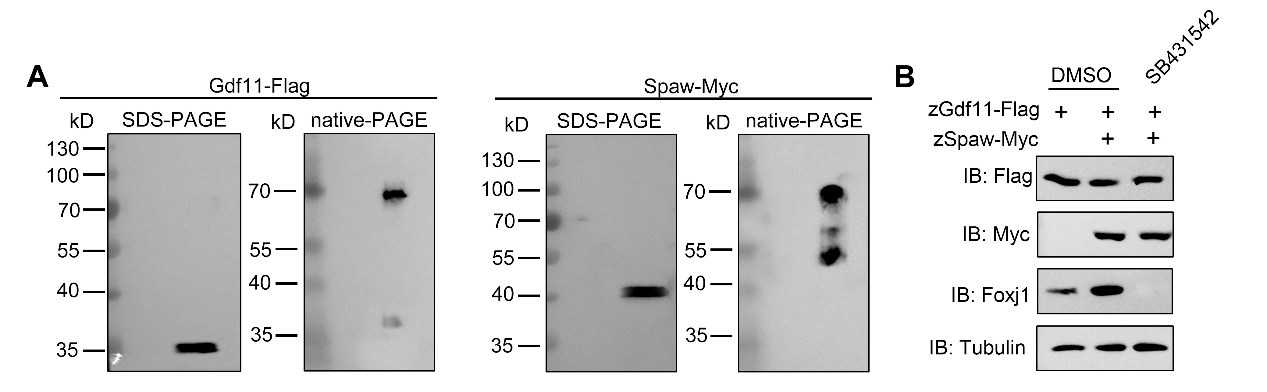


**Figure S4 Gdf11 interacts with Spaw and promotes Foxj1 protein level.** (A) HEK293T cells were transfected with the indicated plasmids encoding Gdf11 and Spaw. Lysates were immunoprecipitated with anti-Flag or anti-Myc antibodies and then immunoblotted with the indicated antibodies. (B) HEK293T cells were transfected with the indicated plasmids and then harvested for western blots against Flag, Myc, Foxj1, and Tubulin antibodies with DMSO or SB431542 treatment.

**Uncropped blots**


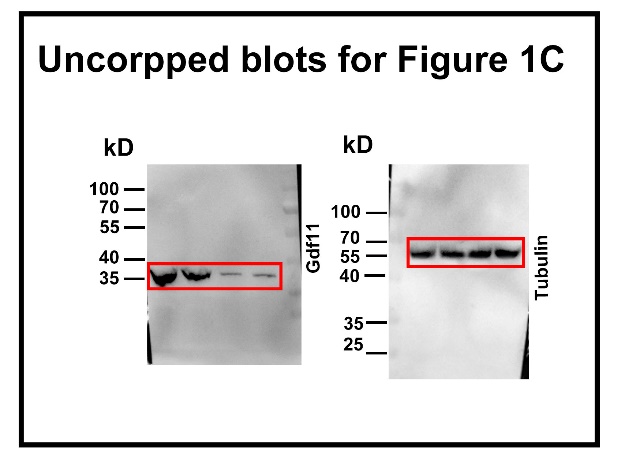


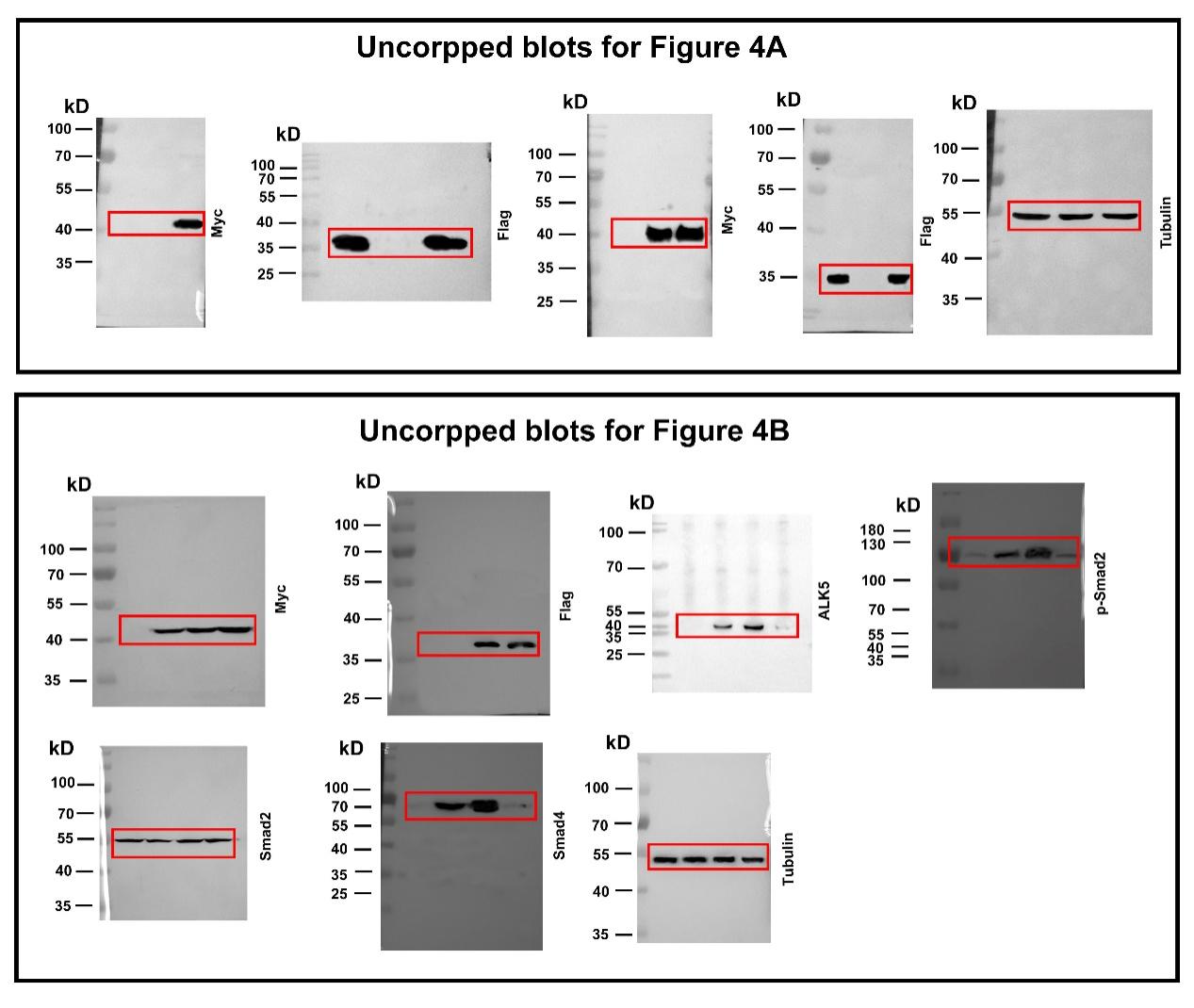


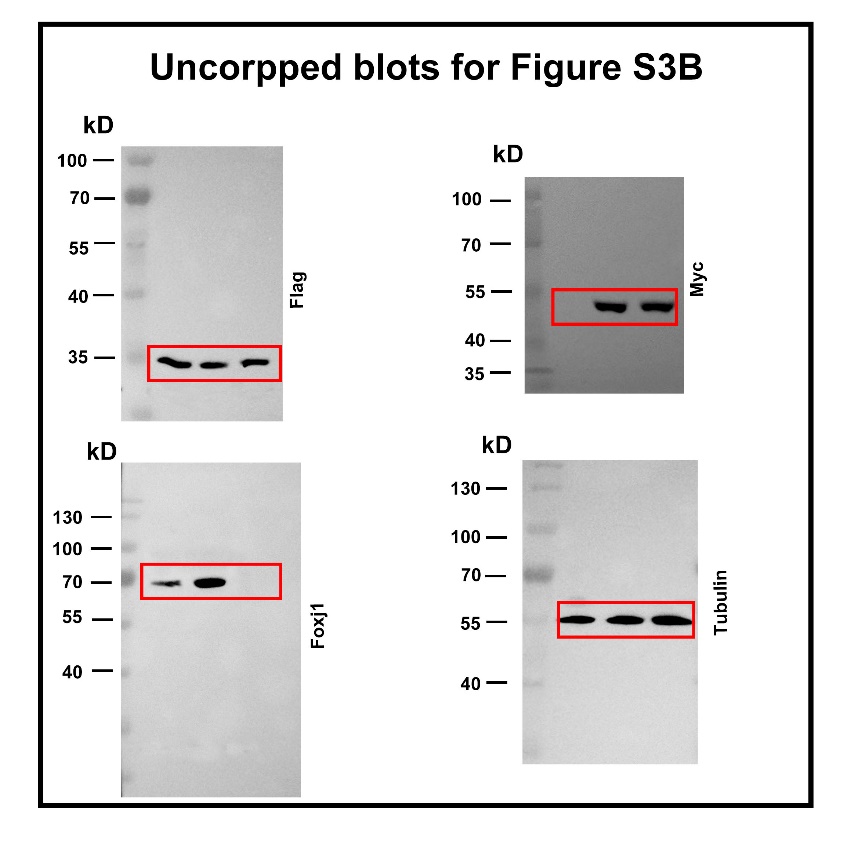

Supplement: Supplementary file 1 — Figure S1. [file CPR-58-e13765-s002.docx]
